# Supplementary material for: Content-rich biological network constructed by mining PubMed abstracts
Source: BMC Bioinformatics. 2004 Oct 8;5:147. doi: 10.1186/1471-2105-5-147 (PMC528731; doi:10.1186/1471-2105-5-147)
Supplement: Additional File 2 — The original results of the above study (non-essential files are deleted to keep the file size under the limit set by BMC bioinformatics). [file 1471-2105-5-147-S2.bz2 › chilibotAdditionalFile2/dip50/137ID7816143E492/html/FOS_JUN.html]

 


 **FOS** and **JUN** 
  
Found 4259 abstracts in PubMed, retrieved 50.  
 

 What does Google say? 
 PDF only 
| .edu only 

---

**Interactive relationship** (e.g. stimulation, inhibition, etc)

**Inhibitory relationship**- Gene expression for  **Fos**  was suppressed at both times, while that for Fosl1 and  **Jun**  was augmented at 12 h and suppressed at 48 h.  Ref: 12875578 Acta Otolaryngol, 2003
- Also, simvastatin reduced NF kappaB and AP 1  [ **JUN** ]  dependent reporter gene activity in TNF alpha treated HT 1080 fibrosarcoma cells and reduced the nuclear levels of p50 NF kappaB, p65 NF kappaB, and the AP 1  [ **JUN** ]  components c  **fos**  and c  **jun**  in HMC.  Ref: 12753293 Kidney Int< MedlineTA>Kidney Int, 2003
**Neutral relationship**- Under conditions of high rhodopsin availability during exposure to light, Fra 2 and, to a minor degree, FosB substitute for c  **Fos**  and enable light induced AP 1  [ **JUN** ]  activity and thus photoreceptor apoptosis.  Ref: 12766089 Invest Ophthalmol Vis Sci, 2003
- Here, the major cis regulatory elements that control IFN gamma inducible NOS2 gene transcription in human lung epithelial cells are identified as composite response elements that bind signal transducer and activator of transcription 1 STAT 1 and activator protein 1  [ **JUN** ]  AP 1  [ **JUN** ] , which is comprised of c  **Fos** , Fra 2, c  **Jun** , and JunD.  Ref: 12788789 Am J Physiol Lung Cell Mol Physiol, 2003
- We show that c  **Fos**  interacts with STAT 1 after IFN gamma activation and the c  **Fos**  STAT 1 complex binds to the gamma activated site GAS element in close proximity to AP 1  [ **JUN** ]  sites located at 4.9 kb upstream of the transcription start site.  Ref: 12788789 Am J Physiol Lung Cell Mol Physiol, 2003
- FGF2 treatment also induced  **fos**  and  **jun**  mRNAs and later increased the nuclear levels of activator protein 1  [ **JUN** ]  AP1  [ **JUN** ] .  Ref: 12815619 Dev Dyn, 2003

**Non-interactive relationship** (e.g. studied together, co-existance, homology, etc.)

- OBJECTIVES To investigate the effects of microcystins on cell cycle and expressions of c  **fos**  and c  **jun** , and explore the potential carcinogenic mechanisms of Microcystins.  Ref: 12760790 Zhonghua Yu Fang Yi Xue Za Zhi, 2003
- The constitutively active mutants of PKC alpha and PKC epsilon also activated c  **fos** , c  **jun**  and cyclin E promoter activity.  Ref: 12794082 J Biol Chem< MedlineTA>J Biol Chem, 2003
- In addition, using an in vitro model of ovarian cancer, we demonstrated that malignant transformation of ROSE cells resulted in alteration of downstream effectors of the EGFR pathway, as exemplified by aberrant expression of p66Shc, c  **Jun** , c Myc, c  **Fos** , Lot1, p21Cip Waf, and cdc25A.  Ref: 12849999 Biochem Biophys Res Commun, 2003
- Since activator protein 1  [ **JUN** ]  AP 1  [ **JUN** ] , a heterodimer of c  **jun**  and c  **fos**  proteins, is known to inhibit binding of AR to ARE by protein protein interaction with AR, the findings in the present study suggest a possible involvement of AP 1  [ **JUN** ]  in the antiandrogenic effects of PAHs acting as AhR agonists.  Ref: 12799773 Arch Toxicol, 2003
- In a Gal4 fusion assay, Pdcd4 specifically inhibited activation of c  **Jun**  and c  **Fos**  activation domains, but did not inhibit activation of JunB, JunD, Fra 1, or Fra 2.  Ref: 12802278 Oncogene, 2003
- Northern blot analysis 30 min after growth factor stimulation showed that c  **FOS**  and c  **JUN**  mRNAs were induced more strongly in neointimal than in medial cells.  Ref: 12808345 J Vasc Res< MedlineTA>J Vasc Res, 2003
- Genes that were commonly down regulated in both PB T cells and Jurkat T cells included CDK inhibitors p16, p19, p27, proapoptotic caspases, and the transcription factors c  **fos**  and  **jun**  B.  Ref: 12808112 Lab Invest, 2003
- These results demonstrate that contextual memory retrieval is associated with de novo synthesis of specific members of the  **Fos**   **Jun**  transcription factor family.  Ref: 12882314 Genes Brain Behav, 2003
- Smad, and 4 and AP 1  [ **JUN** ]  complex c  **fos**  and c  **Jun**  cellular localization were evaluated by immunostaining.  Ref: 12772773 Inflammation, 2003
- The antiandrogenic PAHs elevated mRNA levels of c  **fos**  and c  **jun** .  Ref: 12799773 Arch Toxicol, 2003
- The redox function of Ref 1 involves reduction of oxidized cysteine residues within the DNA binding domains of several transcription factors, including  **Fos**  and  **Jun** .  Ref: 12773568 Mol Cell Biol, 2003
- A nuclear run on assay showed induction of c  **fos**  and c  **jun**  transcripts at the end of the exposure, peaking at 12hr after resuspension of cells in drug free medium.  Ref: 12818361 Biochem Pharmacol< MedlineTA>Biochem Pharmacol, 2003
- The levels of c  **jun**  mRNA and c  **Fos**  protein and mRNA in selenite injected rats also increased more than in the control.  Ref: 12809946 Eur J Pharmacol, 2003
- In these mice context dependent memory retrieval evoked a marked induction of c  **Fos**  and JunB, but not of c  **Jun**  and JunD, in pyramidal CA1 neurons of the dorsal hippocampus.  Ref: 12882314 Genes Brain Behav, 2003
- E2 inhibited constitutive Sp1 and 12 O tetradecanoylphorbol 13 acetate induced AP 1  [ **JUN** ]  transcriptional activities whereas it did not inhibit DNA binding of Sp1 or AP 1  [ **JUN** ]  c  **Fos**  c  **Jun** .  Ref: 12787135 J Invest Dermatol, 2003
- Furthermore, cycloheximide inhibited the increased c  **Fos**  and c  **Jun**  protein expression levels induced by KA in the hippocampus.  Ref: 12788213 Brain Res Bull< MedlineTA>Brain Res Bull, 2003
- To this end, we studied the kinetics of serum stimulated transcription of c  **fos** , c  **jun** , fra 1, egr 1, and cyclinD1 genes, as well as the effects of sodium butyrate, an inhibitor of histone deacetylase activity, on transcription of these genes in normal REF cells and transformants E1A ras.  Ref: 12804589 Biochem Biophys Res Commun, 2003
- Thus, Pdcd4 suppresses tumor phenotype by inhibiting AP 1  [ **JUN** ]  dependent transcription, possibly through inhibiting c  **Jun**  and c  **Fos**  activation.Oncogene 2003 2 712 3720.  Ref: 12802278 Oncogene, 2003
- Western blots for AP 1  [ **JUN** ]  and its signal mediators Erk and JNK showed that expression of  **Fos**  and JNK were decreased by the addition of FZFAT at 300 microg ml.  Ref: 12856862 Am J Chin Med, 2003
- Since the proteins of the  **Fos**  and  **Jun**  families of immediate early genes dimerize to form activating protein AP 1  [ **JUN** ] , the present study was conducted to examine the expression of  **Jun**  transcription factors in schizophrenic and control subjects.  Ref: 12799614 Neuropsychopharmacology< MedlineTA>Neuropsychopharmacology, 2003
- Supershift electrophoretic mobility shift assay indicated that JunB, JunD, and c  **Fos**  components of AP 1  [ **JUN** ]  were particularly affected.  Ref: 12857751 J Biol ChemJ Biol Chem, 2003
- We report that, identically to 17beta estradiol or dihydrotestosterone, but differently from raloxifene, estren alters the activity of Elk 1, CCAAT enhancer binding protein beta C EBPbeta, and cyclic adenosine monophosphate response element binding protein CREB, or c  **Jun**  c  **Fos**  by an extranuclear action of the ER or AR, resulting in activation of the Src Shc ERK pathway or downregulation of JNK, respectively.  Ref: 12782668 J Clin Invest, 2003
- In conclusion, hypothermia decreased cerebral infarction in association with early increases in c  **Fos**  expression and AP 1  [ **JUN** ]  DNA binding activity in peri infarct cortex.  Ref: 12763603 Brain Res, 2003
- IR was found to enhance the expression of c  **Jun**  and, in particular, ATF3, but, in contrast to various other stress stimuli, did not induce the expression of c  **Fos** .  Ref: 12833146 Oncogene, 2003
- These results suggested that selenite induced apoptosis accompanied by the activation of caspase 3 and JNK and the upregulation of c  **jun** , c  **fos** , p53 and p21 WAF1 CIP1 at the early stage of liver regeneration.  Ref: 12809946 Eur J Pharmacol, 2003
- We first assessed the effect of oxidant exposure on AP 1  [ **JUN** ]  c  **Jun**  and  **Fos**  and c  **Jun**  N terminal kinase JNK regulation in MLE12 cells.  Ref: 12842852 Am J Respir Cell Mol Biol< MedlineTA>Am J Respir Cell Mol Biol, 2003
- Both VPA and lithium altered the expression of the early inducible genes for c  **fos**  and c  **jun**  thus promoting the expression of specific proteins.  Ref: 12890311 Int J Neuropsychopharmacol, 2003
- Thus, not the absence of c  **Fos**  per se, but rather impairment of AP 1  [ **JUN** ]  DNA binding is protective against light induced damage.  Ref: 12766089 Invest Ophthalmol Vis Sci, 2003
- In these cell lines, stimulus induced increases in c  **Fos**  protein levels were dramatically attenuated, while c  **Jun**  and CREB levels remained unchanged.  Ref: 12787062 J Neurochem, 2003
- The aim of this study was to examine the effect of hypothermia on expression of c  **Fos**  and c  **Jun** , and AP 1  [ **JUN** ]  DNA binding activity, after transient focal cerebral ischemia in rat brain, and clarify the role of IEGs and AP 1  [ **JUN** ]  after insults.  Ref: 12763603 Brain Res, 2003
- Both oxidants induced c  **Jun**  and  **Fos**  expression, albeit with a different pattern of regulation hyperoxia 95% O2 inducing a biphasic response while H2O2 500 micro M induced a sustained response.  Ref: 12842852 Am J Respir Cell Mol Biol< MedlineTA>Am J Respir Cell Mol Biol, 2003
- CONCLUSION Up regulating the expression of transcript factor such as c  **fos**  and c  **jun**  thus to induce cell abnormal proliferation may be the potential carcinogenic mechanisms of microcystins.  Ref: 12760790 Zhonghua Yu Fang Yi Xue Za Zhi, 2003
- Compared with RPE65Met 450, introduction of the RPE65Leu 450 variant led to increased levels of RPE65 protein, accelerated rhodopsin regeneration, loss of protection against light induced damage, and AP 1  [ **JUN** ]  responsiveness to toxic light doses, despite the absence of c  **Fos** .  Ref: 12766089 Invest Ophthalmol Vis Sci, 2003
- Moreover, by using a competitive electromobility shift assay, we found that AP 1  [ **JUN** ] , a  **Fos**   **Jun**  heterodimer, in HCS 2 8 was capable of binding not only to a typical AP 1  [ **JUN** ]  binding DNA fragment but also to the enhancer fragment of the ctgf gene.  Ref: 12811624 J Bone Miner Metab, 2003
- In addition to shock response genes, such as hsp70 and hsp40, the stress response genes c  **jun** , c  **fos**  and egr 1 were expressed in the early phase after heat shock.  Ref: 12841876 Cancer Sci, 2003
- Effect of intra ischemic hypothermia on the expression of c  **Fos**  and c  **Jun** , and DNA binding activity of AP 1  [ **JUN** ]  after focal cerebral ischemia in rat brain.  Ref: 12763603 Brain Res, 2003
- MATERIAL AND METHODS From cDNA microarray screening of those specimens, several gene clusters, including Nos2 and the transcription factors  **Fos** , Fosl1,  **Jun**  and Nfkb1, were identified as possible upstream regulators of Na transport protein expression.  Ref: 12875578 Acta Otolaryngol, 2003
- Increased c  **Fos**  immunoreactivity in the cortex was observed at 3 h after reperfusion in the HT, but not the NT group, while c  **Jun**  expression was not affected by HT treatment.  Ref: 12763603 Brain Res, 2003
- RESULTS Microarray analysis revealed that the mRNA expression of multiple immune modulators, including krox24, interferon gamma regulating factor 1 IRF 1, monocyte chemoattractant protein 1 MCP 1, interleukin 1beta IL 1beta, CCAAT enhancer binding protein C EBP, p21, c  **fos** , c  **jun** , and pJunB, was significantly increased in obstructed compared to sham operated kidneys all P &lt.  Ref: 12787392 Kidney Int< MedlineTA>Kidney Int, 2003
- Using specific polyclonal antisera against c  **Fos** , JunB, c  **Jun**  and JunD, we tried to identify the candidate transcription factors of the immediate early gene family which may contribute to the molecular processes during contextual memory reconsolidation.  Ref: 12882314 Genes Brain Behav, 2003
- In HUVECs exposed to SS, chromatin within c  **fos**  and c  **jun**  promoters was specifically immunoprecipitated by an antibody against acetylated histone H3 on K14.  Ref: 12805238 Circ Res< MedlineTA>Circ Res, 2003
- Also, GLP 2 produced a significant increase in the mRNAs of c  **fos**  and c  **jun**  when gene expression was determined by Northern blotting.  Ref: 12846833 Eur J Biochem, 2003
- CHOP dimerizes with and inhibits the binding of C EBP related transcription factors to their consensus DNA target sequences and also forms novel complexes with other transcriptional proteins e.g. c  **jun** , c  **fos** .  Ref: 12876286 J Biol ChemJ Biol Chem, 2003
- Effects of microcystins on cell cycle and expressions of c  **fos**  and c  **jun**  < ArticleTitle>.  Ref: 12760790 Zhonghua Yu Fang Yi Xue Za Zhi, 2003
- Our results suggest that the increased expression of the c  **Fos** , c  **Jun** , and phosphorylation of ERK, JNK1, and CaMK II proteins may play important roles in the memory impairment and the cell death in CA3 region of the hippocampus induced by i.c.  Ref: 12788213 Brain Res Bull< MedlineTA>Brain Res Bull, 2003
- Supershift analysis verified the participation of  **Jun**  and  **Fos**  in the complexes.  Ref: 12818361 Biochem Pharmacol< MedlineTA>Biochem Pharmacol, 2003
- For growth stimulation of liver cells by hepatocyte growth factor HGF or transforming growth factor alpha TGFalpha via receptor tyrosine kinases, c  **fos**  c  **jun**  has been considered a point of intersection for cross talk between the different signal transduction pathways.  Ref: 12771026 Carcinogenesis, 2003
- A mixture of c  **Fos**  plus c  **Jun**  antisense oligonucleotides prevented late recovery of downregulated CT and CI, without affecting S1P induction of downregulation.  Ref: 12782628 J Biol Chem< MedlineTA>J Biol Chem, 2003
- Immunohistochemistry assay was applied to detect the expressions of c  **fos**  and c  **jun**  at, 6 hr time point, and cell cycle at 4 hr point were analyzed by flow cytometry respectively.  Ref: 12760790 Zhonghua Yu Fang Yi Xue Za Zhi, 2003
- Transformation of REF cells by oncogenes E1A and cHa ras leads to activation of AP 1  [ **JUN** ]  factor concomitantly with down regulation of c  **fos**  gene transcription.  Ref: 12804589 Biochem Biophys Res Commun, 2003
- The same PKCepsilon inhibitors blocked S1P evoked increases in T cell nuclear levels of c  **Fos**  and phosphorylated c  **Jun**  and JunD after 24 h, but not 1 h.  Ref: 12782628 J Biol Chem< MedlineTA>J Biol Chem, 2003
- Three predominant signaling pathways were identified to be inhibited by esculetin a the activation of p42 44 mitogen activated protein kinase MAPK and the downstream effectors of c  **fos**  and c  **jun**  immediate early genes by means of western and reverse transcription polymerase chain reaction RT PCR analyses.  Ref: 12781342 Biochem Pharmacol, 2003
- Although Ref 1 provides the major AP 1  [ **JUN** ]  reducing activity in murine cells, ref 1 C64A cells retain normal levels of endogenous AP 1  [ **JUN** ]  DNA binding activity in vivo as well as normal  **Fos**  and  **Jun**  reducing activity in vitro.  Ref: 12773568 Mol Cell Biol, 2003
- RESULTS Sustained up regulated expression of c  **fos**  and c  **jun**  were induced by MCE during the experimental period, and 5 6 folds increased expression were observed at 6 hour point after treatment.  Ref: 12760790 Zhonghua Yu Fang Yi Xue Za Zhi, 2003
- Here we addressed two issues i how does transcription of  **Fos**   **Jun**  regulated genes change in the cells lacking  **Fos**   **Jun**  heterodimers.  Ref: 12804589 Biochem Biophys Res Commun, 2003
- First, ACTH coordinately induces  **fos**  and  **jun**  gene families via activation of both ERK1 2 and cAMP PKA pathways, resembling a mitogen.  Ref: 12768542 Microsc Res Tech, 2003
- To test whether introduction of the Rpe65Leu 450 variant can overcome protection against light induced photoreceptor apoptosis in mice without the activator protein AP 1  [ **JUN** ]  constituent c  **Fos** .  Ref: 12766089 Invest Ophthalmol Vis Sci, 2003
- Oncogenes, such as c myc, c  **fos** , and c  **jun** , are also up regulated in this model.  Ref: 12855652 Clin Cancer Res, 2003
- The inhibition of S phase induction was associated with down regulation of c Myc, but not of c  **Fos**  or c  **Jun** .  Ref: 12883011 Proc Natl Acad Sci U S AProc Natl Acad Sci U S A, 2003
- Genes induced independently of p53 included the antiapoptotic cFLIP gene and known stress genes related to the mitogen activated protein kinase pathway and the  **Fos**   **Jun**  pathway.  Ref: 12782583 Cancer Res, 2003
- Together with bZIP proteins of the  **Fos**  and  **Jun**  families, ATF2 constitutes the AP 1  [ **JUN** ]  transcription factor complex.  Ref: 12751796 Biol Chem, 2003
- Several putative transcriptional factor binding sites for nuclear factor of activated T cells 1, AP1  [ **JUN** ]  c  **JUN**  and c  **FOS** , AP2, GABP, OCT1, GATA3, PRE, and C ETS1 were predicted in the promoter region.  Ref: 12816724 Neuro oncol< MedlineTA>Neuro oncol, 2003
- Electrophoretic mobility shift assay revealed that the elevated cAMP potentiated AP 1  [ **JUN** ]  binding activity by enhancing c  **fos**  binding.  Ref: 12750010 Brain Res Mol Brain Res, 2003
- Following a 24 hr exposure to 100& ; mgr ; M oltipraz, elevated steady state levels of mRNA for  **Jun**  and  **Fos**  family members were observed.  Ref: 12818361 Biochem Pharmacol< MedlineTA>Biochem Pharmacol, 2003
- The expression levels of heat shock protein 70 HSP70, c  **jun**  and c  **fos**  genes were remarkably up regulated and those of c myc and poly ADP ribose polymerase PARP were down regulated in bupivacaine treated cells.  Ref: 12820540 J Toxicol Sci, 2003
